# Need for women-centered treatment for substance use disorders: results from focus group discussions

**DOI:** 10.1186/s12954-018-0247-5

**Published:** 2018-08-06

**Authors:** Natasha Elms, Kendra Link, Adam Newman, Susan B. Brogly, for the Kingston House of Recovery for Women and Children

**Affiliations:** 1 Department of Public Health Sciences, Queen’s University, Carruthers Hall, 62 Fifth Field Company Lane, Kingston, Ontario K7L 3N6 Canada; 2 Independent Researcher, 1746 Marian Crescent, Kingston, Ontario K7L 5H6 Canada; 3 Department of Family Medicine, Queen’s University, 220 Bagot Street, Kingston, Ontario K7L 3G2 Canada; 4 Department of Surgery, Kingston Health Science Centre, Queen’s University, Victory 3, 76 Stuart Street, Kingston, Ontario K7L 2V7 Canada

**Keywords:** Substance use treatment, Women, Parenting, Children, Focus group discussion

## Abstract

**Background:**

There are few women-centered treatment programs for substance use disorder. We therefore undertook an exploratory study to better understand the treatment experience, barriers, and facilitators of mothers with substance use disorder.

**Methods:**

We conducted two focus groups with a total of ten women with a history of substance use disorder in Kingston (Canada). Women were recruited from a community program for mothers with substance use disorder. The focus groups were recorded, and the resulting data were transcribed, coded, and thematically analyzed. Barriers, facilitators and treatment needs were identified.

**Results:**

The mean age of the participants was 31.1 years, 30% were currently using substances, and 60% had a child in their care. A key concern for women regarding substance use treatment was the welfare of their child(ren). Agencies charged with child protection were a barrier to treatment because women feared disclosing substance use would result in loss of child custody. In contrast, when agencies stipulated that women must attend treatment to retain custody, they facilitated treatment engagement. Other barriers to treatment included identifying treatment programs and completing admission requirements, wait times, counselor ability to address woman-centered issues, fear, safety, and stigma. Women’s personal motivation for treatment was a facilitator. Suggestions to improve treatment programs included to allow children to accompany their mothers, involvement of peer support, and women-only programs.

**Conclusions:**

This small but novel study provides important data to inform treatment programming for mothers with substance use disorders.

## Background

The ongoing high rate of opioid use is a major public health concern in Canada and the USA. Of the 2.1 million initiators of opioid misuse per year in the USA, 1.2 million (57%) are women [1]. In Ontario (Canada), there was a 16-fold increase in the number of deliveries among women with substance use disorder from 2002 to 2016 [2].

Lack of available substance use treatment has historically been problematic for users wanting to engage in care, and this has been further challenged by the ongoing increases in opioid use [3]. Twenty-seven percent of women with opioid use disorder in Ontario who delivered from 2002 to 2014 did not have a prescription record of opioid agonist therapy [2]. In the USA in 2016, almost nine out of ten young adults and eight out of ten of adults aged 26 or older who needed treatment for illicit drug use did not receive specialty treatment [4]. And in a recent survey of publicly funded residential addiction treatment programs in Ontario, wait times for priority patients (including pregnant women and patients at risk of harming themselves and others) were on average 3 weeks and as long as 6 weeks [5].

Women with substance use face unique concerns accessing treatment including fear of reprisal and loss of children [6, 7]. Such issues can prevent women from accurately reporting substance use patterns and from receiving appropriate medical and psychological care [8]. Studies have suggested that women-centered treatment programs can lead to better treatment outcomes [9]. Yet, few such programs for women exist [1, 10]. We therefore undertook a small exploratory study of mothers with a history of substance use disorder in Kingston, Ontario, to gain insight to the treatment barriers, facilitators, experience, and needs of women.

## Methods

The study took place in Kingston, Ontario, which has a population of 124,000 and a high prevalence of substance use. To be eligible for participation, women had to be 18 years of age or older, identified as having a substance use disorder, willing to talk in a group of peers, and able to provide informed consent. Women were recruited from participants of a local community health center program called Thrive. Thrive is a publicly funded program, which offers counseling, in-hospital and in-home support visits, and parenting support and education for mothers and pregnant women with substance use disorder without cost [11]. Thrive staff identified and invited women to participate in the focus groups. Focus group participants were provided $25 CDN grocery cards as compensation for their time. Taxi vouchers also were provided to facilitate transportation.

Two focus groups were held in private rooms at a community center. The same template was used at each session. It consisted of open-ended questions about substance use treatment experience, barriers, and facilitators to guide the focus group interviews, to clarify points, and to encourage continuous dialogue (Appendix). The focus group questions were developed by two women who work in community services for disadvantaged women. The questions were then reviewed and revised by an experienced physician (the author AN) and medical researcher (the author SBB) and by a mother with a history of substance use. Two women who work in community services for disadvantaged communities and had experience in group dynamics facilitated the focus group discussion (including the author KL). The focus group interviews lasted 35 min (group 1) and 70 min (group 2). Participants provided written informed consent. The study was approved by Queen’s University Health Sciences Research Ethics Board.

Interviews were audio recorded, transcribed verbatim, coded, and thematically analyzed [12]. We began the data analysis using the process of open coding whereby transcripts were read in full and codes were derived from the data. Identified latent and manifest codes were noted and defined in a codebook, which was then used in the second stage of the analysis process. In this stage, each transcript was again read in full, and sections of text were highlighted per the appropriate code; codes were not mutually exclusive. The final stage of the analysis process was thematic separation in which all participant quotations were placed in separate documents according to the selected code. These documents allowed for overarching themes and sub-themes to be identified across, between, and within each group. Two focus group participants then reviewed the results, use of quotations, and manuscript to validate the study findings.

In addition to the focus group data, women completed a short questionnaire on their substance use and treatment histories, parenting responsibilities, and unmet program needs. The questionnaire was developed as part of another research project [13]. Descriptive frequencies including proportions, means, and standard deviations were calculated.

## Results

### Participant characteristics

The first focus group included three women and the second included seven. The characteristics of the women are provided in Table 1. As shown, the mean age was 31.1 years and all women self-identified as white with one also identifying as native. Sixty percent of the women had children in their care. The mean age of the children was 5.4 years. Three women (30%) were using substances on a regular or semi-regular basis at the time of the focus group. Substance use histories included methamphetamines reported by 77.8% of women, marijuana (66.7%), and opioids (44.4%). In terms of substance use treatment, 60% had ever participated in a substance use disorder treatment program, with 50% ever having participated in a treatment program that required an overnight stay. A majority (80%) of women had wanted to attend a treatment program at some point in their life but were unable to. The most common reason for not attending the program was fear of losing their child(ren) (75%), followed by no care for their child(ren) (62.5%). All women responded that they would likely attend a substance use disorder treatment program that would allow women to bring their child(ren).

**Table 1:** Characteristics of ten women with a history of substance use disorder who participated in the focus group discussions

Characteristic	*N* (%) or mean (± standard deviation)
Age of women (years)	31.1 ± 5.2
Race	
White	9 (90.0)
White and Native	1 (10.0)
Number of children in care	
None	2 (20.0)
1	4 (40.0)
2	3 (30.0)
3	1 (10.0)
Mean age of children (years)	5.4 ± 5.0
Ever used substances on a regular/semi-regular basis	10 (100)
Currently using substances on a regular/semi-regular basis	3 (30.0)
Substances frequently used*	
Alcohol	3 (33.3)
Marijuana	6 (66.7)
Opioids	4 (44.4)
Cocaine	3 (33.3)
Methamphetamine	7 (77.8)
Other stimulants	1 (10.0)
Ever been in a substance use disorder treatment program	6 (60.0)
Ever been in an overnight substance use disorder treatment program	5 (50.0)
Ever wanted to enter a substance use disorder treatment program but was unable to	8 (80.0)
Reason did not attend treatment program	
Waiting list	3 (37.5)
Could not afford it	1 (12.5)
No care for my child(ren)	5 (62.5)
Fear of losing my child(ren)	6 (75.0)
Did not know how to go about it	4 (50.0)
Afraid of losing job	1 (12.5)
Abusive relationship	3 (37.5)
Transportation	3 (37.5)
Would attend a local substance use disorder treatment program if child(ren) could also attend and be cared for	10 (100.0)

*Answer missing for one woman

### Barriers to treatment

Analysis of the focus group data showed that a major factor in the women's treatment was concern for the safety and custody of their children. Fear of Children’s Aid Society (CAS) involvement and threat of child(ren) removal were barriers to disclosing substance use and to engaging in treatment for some women. CAS is the organization that helps to protect infants, children, and youth who are experiencing abuse or who are at risk of experiencing abuse, physically, sexually, emotionally, or through neglect or abandonment [14].

CAS finding out [about the addiction] would be a major stressor because you wouldn’t want them knowing, obviously. (Participant 1)

Some women did not seek treatment because they would have to leave their children. The requirement of limited or no outside communication of many residential treatment programs increased the fear of physical separation from their child(ren). This structure often undermined the effectiveness of the treatment.

It is impossible to focus on yourself when you are busy worrying about everything else under the sun. And especially when you are early in your recovery. It is hard enough to focus on anything as it is … and if you are worried about where your kids are and who they are with… it is so hard. (Participant 8)

Additionally, 12-step programs and group counseling were not viewed as child-friendly in content or language. A lack of space for children at these services was a barrier for women without childcare options. It prevented attendance due to no childcare and also not wanting to expose their child to issues discussed in the meetings. This lack of childcare generated creative solutions.

My daughter has turned into the NA [Narcotics Anonymous] babysitter. She takes the kids down into the basement and does colouring with them, or goes outside and plays. (Participant 5)

Even after a woman decided to attend residential treatment, fear was a major barrier to treatment entry. Fear of the unknown treatment program coupled with fear of treatment being unsuccessful was a major deterrent for women especially when their child(ren) must be left behind.

Well I know it is treatment, but I don’t know what I’m going to be doing in treatment or what exactly I can expect out of it. […] I bet once you’re there you’re probably fine, but it is about going. (Participant 3)

Women discussed counselor competence as a barrier to treatment success. Women were discouraged by counselors who had a lack of understanding or empathy for their gender-specific issues.

I have women-based issues, and I thought I was in a safe place. Oh no, no, no. … I felt that the counsellors were not able to deal with what I was going through at all. (Participant 8)

The potential for ex-partners and volatile men to attend outpatient programs and the perceived lack of safety was another barrier. This furthered the perception of an environment hostile towards children and women as described by participant 4.

I wanted to go to a meeting and there was an altercation in the parking lot. Guys were throwing fists. And it is like, I have my baby with me, and now this makes me feel not comfortable and not wanting to come here because you never know who is going to show up. [… The ideal type of environment would be] Having a safe drop-in place where you could go with your kid and talk without having to worry about some unsavoury language that you would find at some meetings. You know, something that you would feel comfortable bringing your kids to, no matter what age they are. (Participant 4)

Stigma was another pervasive barrier. Stigma generated shame and diminished the woman’s self-esteem and willingness to seek and continue with treatment as noted by many focus group participants.

It is hard with the stigma. Because the second that you ask for help, they’re like ‘Oh, you’re a parent and you are an addict’. (Participant 8)

It is shamed-based, you know. You are made to feel ashamed for making poor choices and you have to carry that for the rest of existence. (Participant 4)

The process of identifying treatment programs, completing forms, and ensuring that providers received required documentation often was the responsibility of the woman and was a potential barrier to treatment. Two women noted how complex the process was.

I don’t even know where I’d begin. I don’t even know where I would start to outreach to. (Participant 1)

The resources are not shining and blinking lights. You have to dig around to find out what is even available… and it is a pain. It shouldn’t have to be this hard to get help. (Participant 6)

Similarly, there was an identified need for increased clarity on what the treatment programs involved.

I want to know more about what is happening in treatment. What happens when I am there, the schedule, and what am I going to be working on? What are we going to be doing? (Participant 2)

To increase awareness of available programs, participants discussed the value of using social media, especially Facebook, and an easily searchable website to connect individuals with appropriate services in their area. Providing flyers in common community areas such as grocery stores, coffee shops, and city buses and in more targeted areas such as family court, hospitals, harm reduction services, and doctor’s offices also was suggested.

Wait times associated with treatment facilities and the requirement of some programs to complete a patient assessment further complicated treatment entry. These barriers sometimes resulted in a missed opportunity for change.

You have to get an assessment done before you can go to treatment. It is ridiculously difficult. […] you are going to wait 11 months for an assessment. You can be dead by then. (Participant 6)

When you want help, you want it now. (Participant 6)

### Facilitators of treatment

When discussing facilitators of treatment (Table 2), women often mentioned personal motivation and hope for moving past substance use. The choice to seek and continue treatment was most often motivated by wanting what was best for their child(ren) and to protect their child(ren) from unsafe situations including CAS, foster care, drug exposure, violence, and ex-partners. Women’s hope for a better future for themselves and their child(ren) was a facilitator.

I have a daughter. […] If I continue then she would […] eventually get into the system. And then she might turn out to be the same way. And that is on me. (Participant 6)

I was very fortunate; my child was with family friends […] he got to be with a loving family. So, but I am sure that if that hadn’t happened, he would be in the system right now. (Participant 4)

**Table 2:** Barriers and facilitators that women experience at each stage of substance use treatment services that emerged from focus group discussions

	Treatment engagement	Treatment retention	Ongoing recovery
Barrier			
Children’s Aid Society (CAS)	Fear of CAS involvement and child(ren) removal prevents disclosing dependency and seeking treatment		
Fear	Losing their child(ren) to CAS Physically getting to the treatment facility Unknowns of the treatment process Being alone Running into ex-partners Stigma from being a mother with addiction Treatment will be unsuccessful	Being alone Running into ex-partners Stigma from being a mother with addiction	Unknowns of the treatment process Running into ex-partners Stigma from being a mother with addiction
Wait times	Services unavailable when women are ready		
Admittance Requirements	Paperwork and other requirements to enter treatment often must be completed/organized by the woman		
Counselors		Staff that cannot effectively engage in women-centered issues discourages continued engagement and does not meet needs	Staff that cannot effectively engage in women-centered issues discourages continued engagement and does not meet needs
Childcare	Treatment requires women find suitable caregivers so they can leave their families, while limiting the contact they have with their child(ren) during this process	Women without alternative childcare options cannot easily access these services; services not child-friendly (in content or language); no physical space for children	Women without alternative childcare options cannot easily access these services; services not child-friendly (in content or language); no physical space for children
Safety	Potential participants (i.e., ex-partners or volatile men) erode the perceived safety, and dissuade women from attending services alone or with their child(ren)	Potential participants (i.e., ex-partners or volatile men) erode the perceived safety, and dissuade women from attending services alone or with their child(ren)	Potential participants (i.e., ex-partners or volatile men) erode the perceived safety, and dissuade women from attending services alone or with their child(ren)
Stigma	Stigma from health care workers and society generates shame which diminishes self-esteem and willingness to seek treatment	Stigma from health care workers and society generates shame which diminishes self-esteem and willingness to continue treatment	Stigma from health care workers and society generates shame which diminishes self-esteem and willingness to continue recovery
Facilitator			
Children’s Aid Society (CAS)	CAS requirements and/or fear of loss of custody	CAS requirements and/or fear of loss of custody	CAS requirements and/or fear of loss of custody
Hope	Hope for a better and safer future for themselves and their child(ren)	Hope for a better and safer future for themselves and their child(ren)	Hope for a better and safer future for themselves and their child(ren)

For women who disclosed their substance use, CAS requirements for the child(ren) to remain in the woman’s custody was a facilitator of treatment engagement.

I had CAS tell me that if I relapsed one more time that they were going to take my children and I would never see them again… and that was it. So, it was time to make a decision and it was time to get some help. (Participant 5)

The women described several elements of successful treatment programs they had experienced. Key among these was an established program structure and participant routine for program engagement. Opportunities to learn real life skills, to develop responsibility, and to show leadership were viewed as successful and useful. Women discussed the value and importance of peer support. This facilitated learning and a sense of pride and responsibility.

We also had, like, peer-counsellors. It was like a thing where everyone voted. […] It was something to strive for. (Participant 4)

Rapport and positive relationships between participants and treatment staff were identified as important. Some women felt that staff experience with substance dependence contributed to the women’s perceived sense of program connectedness and its effectiveness.

It is so important to work with someone that has been through it… because you know that they have done the same things, it makes it so much easier to talk and open up with them. It just makes… everything easier. (Participant 7)

It’s all about the fellowship and just knowing that you have people there that are knowing what you are going through. (Participant 6)

Personalized treatment was also mentioned. This encompassed consideration of the woman’s goals when devising a treatment plan, as opposed to the one-size-fits-all approach that some women experienced. In addition, programs geared to the use and sequelae of specific substances were deemed important. Most women did not attend treatment programs where drug of choice was considered. Programs were sometimes ineffective and did not always meet patient needs.

[successful personalized treatment would include] Time to work on the things you need to work on. Really as much time as you need. Every person is different, right? So every person would need a different amount of time [in treatment]. (Participant 2)

I have heard people be in treatment for 30 days, and then it makes me wonder if it is really beneficial. Because it took me 90 days for my head to come clear. (Participant 5)

Well it depends on the drugs too, right? If it is opiates, then definitely 90 days, probably longer. But crystal meth? You only really need, what is it? A week? Psychologically yes, but just a week it is out of your system. (Participant 4)

## Discussion

This small, exploratory qualitative study provided insight to the unique treatment barriers and facilitators of mothers with substance use disorders. Predominant among the barriers were concerns regarding children. Many residential treatment programs require women to physically leave their child(ren) and restrict contact while in treatment; thus, women must find caregivers for their child(ren) while in treatment or surrender their child(ren) to foster care. Other studies also have identified that the risk of losing child custody is an important barrier to accessing addiction treatment among women [6, 13, 15]. The detrimental effect of child welfare programs in the context of women’s recovery has also been previously noted [15].

Of the 1004 publicly funded treatment programs in Ontario (i.e., community treatment programs, withdrawal services, residential treatment, peer support programs, addiction counseling, etc.), 151 (15.0%) are specific for women. Eighteen (36.7%) of the 49 residential treatment programs are for women only [16]. There are only two publicly funded residential treatment programs in Canada that allow women to attend with their children [17, 18]. Neither is located in Ontario and thus are unavailable to women in the province. Mothers and pregnant women in Kingston are referred to the single program for parenting women [11]. Even in outpatient treatment and group programs such as NA, women face childcare issues. Only 6.5% of outpatient treatment programs across the USA had childcare services for women [19].

Despite the paucity of women-centered treatment programs, emerging data support better outcomes for women who participate in women-centered programs. In a randomized study of a women-only recovery group compared with a mixed sex recovery group, those in the women’s group had greater continued reductions in substance use at 6 months [9]. A systematic review also suggested that integrated treatment programs for women with substance use disorders and their children (i.e., those that include on-site pregnancy, parenting, or child-related services with addiction services) may be associated with better parenting outcomes [20].

Stigma associated with substance use disorder in mothers was a barrier faced by women in our study as has been reported by others [7, 21–23]. Stigma in society around parenting, substance use, and poverty is common [6]. In another qualitative study it was identified that women-only programs facilitated discussion of topics underlying women’s addiction including abuse, physical health, childcare, and self-image [23]. Similarly, in our study, women identified that addiction counselors who could understand their women-based issues were critical to treatment effectiveness. Other women noted that personal experience with addiction better engaged and connected them while in treatment, and facilitated treatment success.

Fear of treatment and a lack of knowledge of what it involved were also identified in our study. Women discussed difficulties identifying treatment programs and completing the necessary documentation and intake requirements. Others have also found that transportation and logistical issues are barriers to treatment among women [15]. A peer support program for women was suggested for treatment engagement and for long-term recovery in our study. This also was recommended by women attending a women-centered program in Vancouver to both facilitate transition of new participants and to maintain connections and ongoing recovery support for women who completed the program [6]. Such avenues should be considered.

Women expressed concerns of personal safety being around abusive ex-partners who also attend treatment programs. Women who experienced intimate partner violence were particularly fearful. The safe atmosphere of women-only groups has been identified by others as important in women’s treatment and recovery [23]. Programs that meet these unique needs of women with substance use disorders should be developed.

Limitations of our data include the small study size and the recruitment of women already engaged in a program for mothers with substance use disorder. Particularly disadvantaged women who may have additional barriers to care may not be represented. The majority of the participants identified as white, and while this reflects our underlying population in Kingston, it may not reflect other populations of substance-using women in Ontario [24]. In addition, none of the women who participated were pregnant.

## Conclusions

In summary, childcare and safety were common barriers to treatment for women with substance use disorder identified in this exploratory qualitative study. Women viewed peer support and targeted programs as needed services. Our data, in addition to that of other studies, support the need for women-centered services to reduce the harms of substance use and to increase a woman’s ability to provide a safe and healthy life for herself and her child(ren).

## Appendix

### Focus group discussion questions

- For those of you that sought treatment, how did you go about this?
- What stands out for you as major barriers to receiving treatment for substance use?
- How did being pregnant affect seeking treatment?
- How does having children affect seeking treatment?
- What has been your experience while in treatment centers?
- What resources or support would be most helpful to you and your family?
- What would be the best way to reach families like yours with information? Who would you trust to bring you information?
- Is there anything else that you think it is important for us to know?
- Are there things in this community that helped you and others in your recovery? What has been most helpful to you? What were the challenges?

## Abbreviations

CAS: Children’s Aid Society
NA: Narcotics anonymous
